# Prevalence of mild cognitive impairment and its association with malnutrition in older Chinese adults in the community

**DOI:** 10.3389/fpubh.2024.1407694

**Published:** 2024-08-14

**Authors:** Ling-ying Wang, Zi-yi Hu, Hong-xiu Chen, Chun-fen Zhou, Xiu-ying Hu

**Affiliations:** ^1^Department of Critical Care Medicine, West China Hospital, Sichuan University/West China School of Nursing, Sichuan University, Chengdu, China; ^2^Innovation Center of Nursing Research and Nursing Key Laboratory of Sichuan Province, West China Hospital, Sichuan University/West China School of Nursing, Sichuan University, Chengdu, China; ^3^Department of Nursing, West China Hospital, Sichuan University/West China School of Nursing, Sichuan University, Chengdu, China; ^4^Mental Health Center, West China Hospital, Sichuan University/West China School of Nursing, Sichuan University, Chengdu, China

**Keywords:** mild cognitive impairment, older adults, malnutrition, community, prevalence

## Abstract

**Objective:**

This study aims to characterize the prevalence and associated factors of cognitive impairment in older adults within Chinese community settings.

**Background:**

Research exploring the interrelation between malnutrition and cognitive impairment in the older adult community-dwelling population is scarce. The impact of nutritional status on cognitive function in aging adults has not been definitively established.

**Methods:**

A cross-sectional survey was conducted in one urban and one rural community in Chengdu, China, from October 2022 to March 2023. The sample included 706 older adults. Logistic regression was utilized to determine independent risk factors for mild cognitive impairment (MCI).

**Results:**

The study found a significant prevalence of MCI at 32.0% among the older adult population. Among those suffering from malnutrition, 55.6% were affected by MCI. The logistic regression analysis indicated that malnutrition risk (OR = 2.192, 95% CI 1.431 to 3.357, *p* < 0.001), rural residence (OR = 1.475, 95% CI 1.003 to 2.170, *p* = 0.048), age (70–79 years old; OR = 2.425, 95% CI 1.611 to 3.651, *p* < 0.001; ≥80 years old: OR = 4.773, 95% CI 2.571 to 8.859, *p* < 0.001), male (OR = 1.584, 95% CI 1.085 to 2.313, *p* = 0.017), middle education level (OR = 0.986, 95% CI 1.627 to 5.482, *p* < 0.001), and ADL dependence (OR = 1.810, 95% CI 1.158 to 2.827, *p* = 0.009) were significantly associated with the occurrence of MCI.

**Conclusion:**

The findings indicate a widespread occurrence of MCI in community-dwelling older Chinese adults. The association between malnutrition, as measured by the Mini Nutritional Assessment-Short Form (MNA-SF), and cognitive decline is evident. Older adult individuals with nutritional risk, advancing age, rural residence, male gender, moderate education, and ADL dependency are at increased likelihood of developing MCI. Longitudinal research is needed to clarify the temporal relationships between MCI, demographic factors, and whether improvements in nutritional status or ADL can reduce the incidence of MCI in this population.

## 1 Introduction

As the global population ages, the imperative of older adult care has garnered international concern. The 2020 census data from China reveals that individuals aged 60 and above constituted 18.7% of the population, a figure projected to rise to 26% by 2050, surpassing the proportion in most European nations (1). The escalation of societal aging across various regions globally has seen a concomitant rise in malnutrition among the aged, an issue not confined to developing nations (2). Malnutrition exacerbates diseases related to advancing age and is, concurrently, a byproduct of the senescence process (3), exhibiting higher prevalence rates among the older adults as compared to younger demographics (4, 5). Age-associated risk factors for malnutrition include diminished gustatory and olfactory acuity (6), gastrointestinal dysfunctions (7), and dental health complications (8).

Concurrent with the aging demographic shift, cognitive impairment has emerged as another significant public health challenge. A 2021 meta-analysis (9) reported a 15.4% prevalence of cognitive impairment among Chinese seniors over 55 and this proportion varies depending on the diagnostic criteria used. Mild cognitive impairment (MCI) refers to a subjective and objective decline in the functional level of one or more cognitive dimensions compared to the past. Still, it does not seriously affect daily instrumental activities and does not lead to mental or other psychological diseases (10). Research has shown that MCI is associated with advanced age, sex, family history, low education level, living alone, low life satisfaction, less engagement in mental activities, low intake of fruits and vegetables, not practicing calorie restriction and the presence of cardiovascular risk factors such as hyperlipidaemia, hypertension, stroke, and coronary heart disease (11–15). Cognitive impairment heightens the risk of progressive conditions like dementia or Alzheimer’s disease (16), with subsequent comorbidities including disability, frailty, and mortality, imposing considerable strain on patients, families, and society at large (17).

Aging is associated with various degenerative diseases, including cognitive impairment, cancer, sarcopenia, dementia, and various chronic non-communicable diseases (18–21). These diseases are closely related to malnutrition, physical weakness, and cognitive impairment. Adequate to high amounts of protein (22) and at least 16 micronutrients, i.e., beta-alanine, calcium, creatinine and so on, have been reported to improve musculoskeletal health and/or cognitive function in older people (23). However, in efforts to improve the health of the adult population, nutritional deficiencies often need more attention (24). Insufficient nutrition in older adults may damage or affect their physical and cognitive function levels (25). Several studies have found a strong relationship between physical frailty and cognitive impairment, suggesting that there may be a common mechanism between these conditions, which may include malnutrition (26–28). In Lee et al.’s (29) study of older people living in particular housing, the difference in nutritional status between older people with normal and reduced cognitive ability was examined. This study showed that older adults with cognitive impairment were more likely to be malnourished in comparison to those with average cognitive ability.

Fortunately, early detection of MCI harbors the potential for reversal, allowing for timely intervention and possibly restoring normal cognitive function (13, 30). A multidomain intervention that includes four intervention components, diet, exercise, cognitive training, and vascular risk monitoring, could improve or maintain the cognitive functioning of these individuals (31). Despite the significant implications, research into the interplay between malnutrition and cognitive impairment in community-dwelling older adult is scant, and the influence of nutritional status on cognitive health in aging individuals remains to be substantiated. Accordingly, this study elucidates the prevalence and contributory factors of cognitive impairment among older adults in Chinese communities.

## 2 Methods

Using a convenient sampling method, a cross-sectional research design was employed for this investigation, encompassing both an urban and a rural community in Chengdu, China, from October 2022 to March 2023. This study used community-dwelling older adult as the research subjects, and survey letters were distributed to all older adult individuals. During this period, all older adults who met the following criteria were included in this study: (a) age ≥ 60 years and (b) agreed to cooperate after the researchers explained the research purpose. Exclusion criteria: mental disorders (Alzheimer’s disease, Schizophrenia), serious and end-stage diseases [life expectancy <12 months, with an established life-limiting condition or in receipt of end of life palliative care services (32)].

Ethical approval for the study was granted by the Ethics Committee of West China Hospital, Sichuan University, in 2022 (Ethics No. 861). We duly obtained informed consent from all participants. After informed consent, the research team proceeded with data collection. We invited 900 older adult individuals, and ultimately 706 of them responded.

We designed a questionnaire to collect sociodemographic data from the participants. The variables included age, sex, height, weight, marital status (married or non-married), residence (urban or rural), and educational level (low: education years <9 years; middle: education years between 9 and 12 years; high: education years >12 years). We calculated body mass index (BMI) by the equation below: BMI = weight (in kg)/height^2^ (in m^2^). Globally, the average BMI varies significantly among different countries (33, 34). Using a lower BMI threshold than the WHO international standard or the improved Asian BMI standard to define overweight and obesity can better illustrate the current BMI status of Chinese people (35, 36). Refer to the Chinese criteria for adults, individuals were divided into underweight (BMI less than 16.5 kg/m^2^), normal weight (BMI between 18.5 and 23.9 kg/m^2^), overweight (BMI between 24 and 27.9 kg/m^2^), and obese (BMI greater than 28 kg/m^2^) groups (37).

The Chinese version of the Mini-Mental State Examination (MMSE), a 30-point questionnaire, was employed to evaluate cognitive function. The Chinese version of the MMSE is an adapted version of the scale developed initially by Folstein (38). Its reliability and validity have been thoroughly verified (39–41). The MMSE is the most widely used cognitive screening test by physicians and researchers for general cognitive evaluation (42, 43). This widely utilized tool encompasses various tasks assessing orientation to time and place, memory recall, arithmetic calculation, language abilities, and basic motor skills (38). Mild cognitive impairment (MCI) thresholds were determined using education-adjusted cut-off scores: ≤19 for non-literate individuals, ≤22 for those with elementary education, and ≤ 26 for participants with middle school education or higher (43).

The Barthel Index (BI), conceived by Mahoney (44) and Barthel in 1965, assessed ADL competencies, including feeding, bathing, grooming, dressing, bowels and bladder, toilet use, transfers, mobility, and climbing stairs. Individual scores of the 10 items range from 0 (total dependence) to 100 (complete independence). Hou offered, translated and introduced the BI survey in Chinese (45). We divided the individuals into complete independence (BI scores = 100) and dependence (BI scores<100) groups (45).

Nutritional status was gaged via the Mini Nutritional Assessment-Short Form (MNA-SF). The MNA-SF is divided into six parts: weight loss during the past 3 months, appetite during the past 3 months, mobility, psychological stress, neuropsychological problems, and BMI or calf circumference (46). Each section is scored on a scale of 0 to 2 or 3, and the total score ranges from 0 to 14 points (47). The MNA-SF has been validated and demonstrates reasonable specificity and sensitivity for diagnosing malnutrition, especially in older adults (48, 49). Participants were categorized into three groups based on total scores: those with an MAN-SF score > 11 were defined as normal nutritional status, those with an MNA-SF score between 8 and 11 were defined as malnutrition risk, and those with an MNA-SF score < 8 were defined as malnutrition (50). The MNA-SF has been validated among older adult individuals in China and has extraordinary test characteristics (51, 52).

We conducted categorical data analysis via multivariate logistic regression to investigate the association between malnutrition and cognitive impairment. A *p*-value of <0.05 was considered statistically significant. We computed odds ratios (ORs) with 95% confidence intervals (CIs). All analyses were executed using SPSS software, version 24.0.

## 3 Results

Of the 706 participants of this study, 403 (57.1%) were male, and 303 (42.9%) were female. Individuals aged 80 years or older constituted 14.2% of the sample. Those with a low education level represented 61.8%, while 56.5% resided in urban communities. The majority, 88.5%, were married; underweight participants comprised 4.7%.

**Figure 1:**
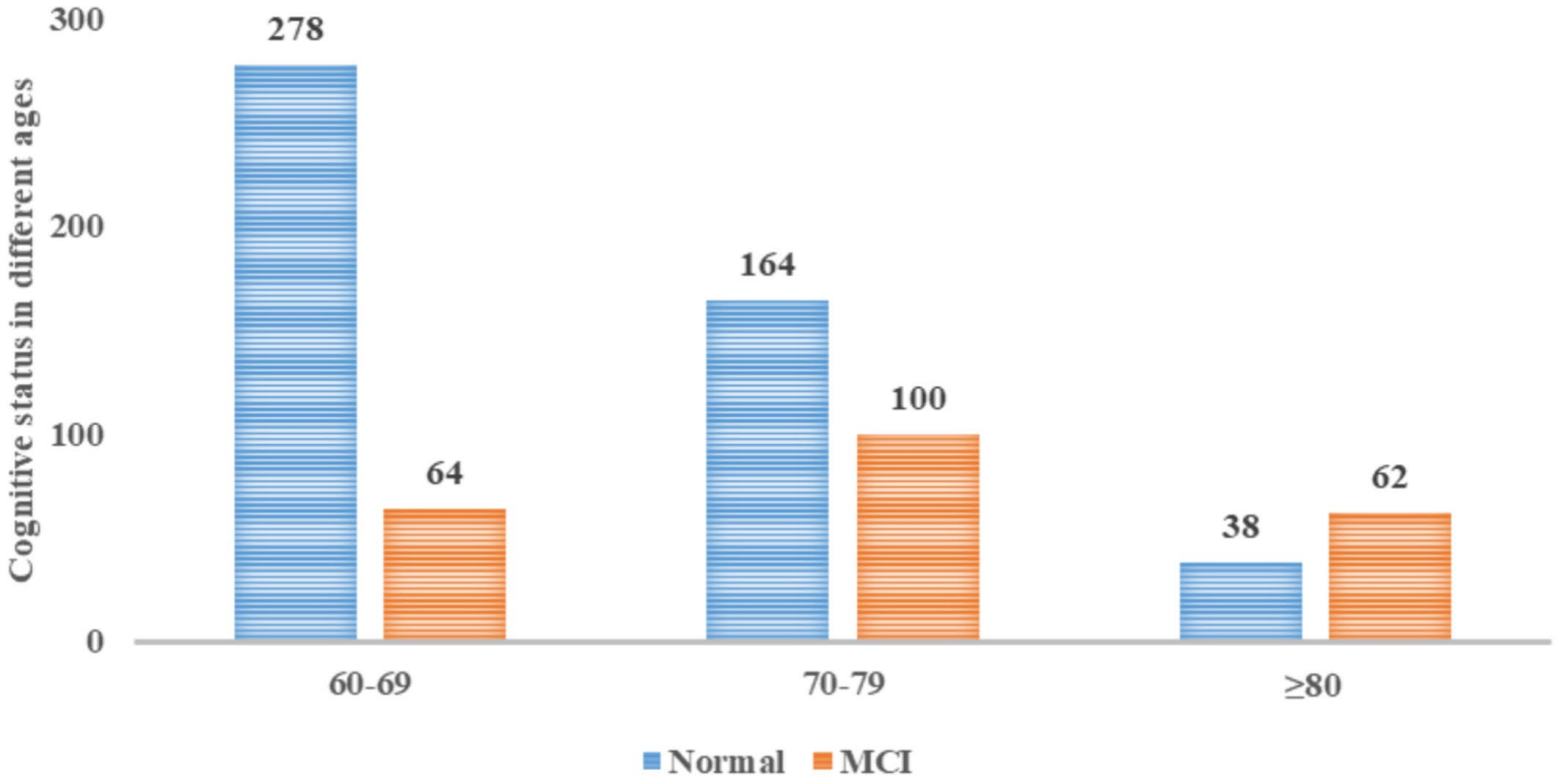
Cognitive status in different ages.

Cognitive status, assessed using the MMSE, indicated that 68.0% of participants presented with normal cognitive function; however, 32.0% (226 individuals) exhibited cognitive impairment. Notably, the old-older cohort (≥80 years) had a 62% incidence of cognitive impairment, significantly surpassing that of the younger groups, as depicted in Figure 1.

**Figure 2:**
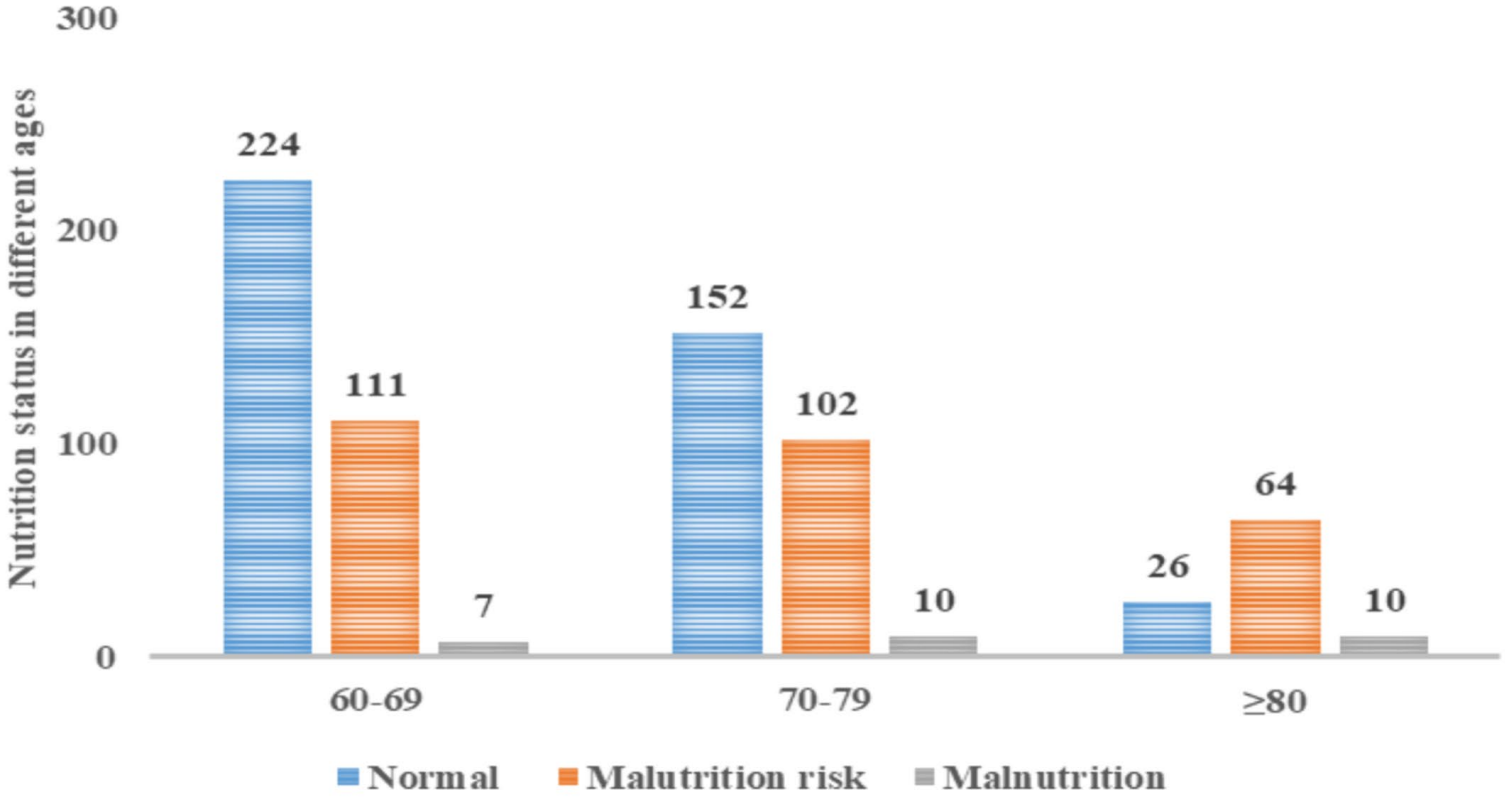
Nutrition status in different ages.

Nutritional assessment through the MNA-SF revealed that 56.9% had satisfactory nutritional status, 39.2% were at malnutrition risk, and 3.8% were malnourished. A direct correlation between advancing age and malnutrition prevalence was observed. Specifically, in the group aged 80 and above, 10.0% were malnourished, and 64.0% were at malnutrition risk (Figure 2).

**Figure 3:**
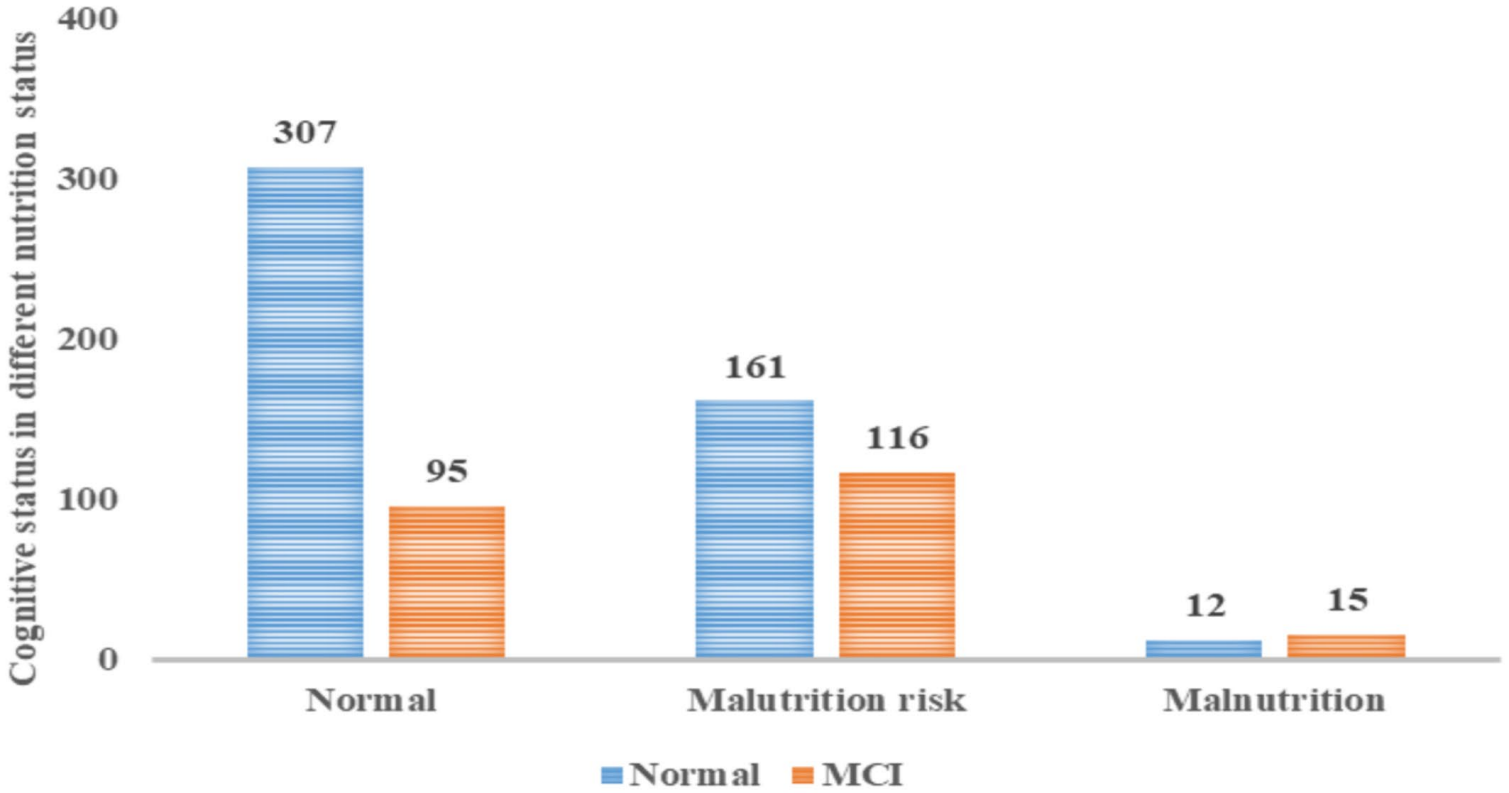
Cognitive status in different nutrition status.

Figure 3 illustrates that 23.63% of malnourished seniors had cognitive impairment, 41.88% at risk of malnutrition were similarly affected, and among the 27 malnourished participants, 15 (55.6%) had MCI.

Logistic regression analysis detailed in Table 1 identified significant associations between MCI and several variables: malnutrition risk (OR = 2.192, 95% CI 1.431 to 3.357, *p* < 0.001), rural residence (OR = 1.475, 95% CI 1.003 to 2.170, *p* = 0.048), age (70–79 years old: OR = 2.425, 95% CI 1.611 to 3.651, *p* < 0.001; ≥80 years old: OR = 4.773, 95% CI 2.571 to 8.859, *p* < 0.001), Male (OR = 1.584, 95% CI 1.085 to 2.313, *p* = 0.017), middle education level (OR = 0.986, 95% CI 1.627 to 5.482, *p* < 0.001), and ADL dependence (OR = 1.810, 95% CI 1.158 to 2.827, *p* = 0.009).

**Table 1:** Logistic regression model of risk factors for MCI among older adults in Southwest China.

Variable	OR	95% CI		*p* value
		Lower	Upper	
Nutrition status				**0.001**
Normal	Ref.			
Malnutrition risk	2.192	1.431	3.357	**<0.001**
Malnutrition	2.462	0.965	6.277	0.059
Residence				
Urban	Ref.			
Rural	1.475	1.003	2.170	**0.048**
Age (years)				**<0.001**
60–69	Ref.			
70–79	2.425	1.611	3.651	**<0.001**
≥80	4.773	2.571	8.859	**<0.001**
Sex				
Female	Ref.			
Male	1.584	1.085	2.313	**0.017**
Education level				**0.001**
High	Ref.			
Low	1.618	0.929	2.820	0.089
Middle	2.986	1.627	5.482	**<0.001**
Marriage				
Married	Ref.			
Non-married	1.534	0.891	2.640	0.122
BMI				0.098
Obesity	Ref.			
Underweight	1.003	0.362	2.779	0.995
Normal weight	0.571	0.298	1.097	0.093
Overweight	0.905	0.481	1.704	0.758
ADL				
Complete independence	Ref.			
Dependence	1.810	1.158	2.827	**0.009**
Constant	0.073			**<0.001**

MCI, mild cognitive impairment; BMI, body mass index; ADL, activity of daily living; Ref, reference. Bold values means *p* < 0.05.

## 4 Discussion

This study aimed to elucidate the relationship between cognitive impairment and its related factors among older adults in Chinese communities, revealing a significant association with nutritional status. Malnutrition, alongside residence, age, sex, education level, and ADL dependency, emerged as critical correlates of cognitive decline.

Nutritional assessment using the MNA-SF indicated that 56.9% of the participants were well-nourished, 39.2% were at risk of malnutrition, and 3.8% were malnourished. The burden of inadequate nutrition was particularly pronounced in individuals aged 80 years and older, with 74.0% being malnourished or at risk. These data are comparable to those in studies with large samples. For example, a meta-analysis involving community-dwelling older adult persons showed that 5.8% of subjects were malnourished, and 31.9% were at risk of malnutrition (53). Notably, the heightened malnutrition risk in participants aged 80 and above underscores the need for greater nutritional vigilance, significantly since aging and associated frailty elevate the risk of disability and functional limitations, potentially precipitating malnutrition. Therefore, malnutrition is pervasive in older adults, and medical staff should pay more attention to the nutritional status of those aged 80 years and older.

Cognitive function was assessed with the MMSE, revealing that 68.0% of participants had normal cognition, whereas 32.0% (226 individuals) experienced cognitive impairment. In our study, the prevalence of MCI was higher than that in a previous study in India (54), which reported a prevalence rate of 18.6%. Another meta-analysis (55) found that the global prevalence of MCI among community-dwelling individuals aged 50 years and older was 15.56%, which was lower than our study’s. Deng’s (9) study found that the prevalence of cognitive impairment among Chinese adults aged 55 years and older was 15.4%. The varying prevalence rates of cognitive impairment suggest that with increasing attention to cognitive aging, cognitive testing has become more common, and cognitive symptoms have been better recognized. The difference may be because our study included more individuals aged 80 years and older, as the prevalence of MCI increases with age. Besides age, the difference in prevalence rates can also be explained by education level. In our study, the education level was relatively low. It has been reported that higher education levels are associated with better cognitive function and slower cognitive decline in older adults (56). Notably, our findings showed a staggering 62.0% incidence of cognitive impairment in those aged 80 and above. Previous studies have shown that in individuals 80 years and older, at least 50% of people, even those who appear healthy, have elevated levels of brain amyloid-β protein (57). The accumulation of amyloid-β protein leads to cognitive decline and makes older adult individuals (aged 80 years and older) more prone to cognitive impairment. Hence, the cognitive health of older adults, mainly those aged 80 and above, presents a grim picture and warrants immediate and concerted intervention efforts.

The association of malnutrition, residency, age, sex, education level, and ADL with MCI in older adults remained significant after adjusting for confounders, paralleling Cong’s findings (58). Cong’s study found that overall, MCI was associated with demographic factors such as age, educational level, famer occupation, non-alcohol consumption, and stroke (58). Another study by Huang (59) found that male sex and higher educational level were associated with an elevated risk of dementia among younger MCI patients in Taiwan. The difference may be because the education level is generally higher among older adult males in Taiwan, and the elevated risk faced by individuals with higher educational levels might be explained by this gender difference (60). The declines in ADL caused by cognitive impairments hinder MCI patients’ independent and safe daily lives (61). We also found that the MCI prevalence in disabled older adults was 1.810 times that of self-cared older adults, which was consistent with the study by Santos (62), which demonstrated that cognitive functions were related to i-and b-ADLs in people with MCI.

Our analysis revealed that the likelihood of MCI in older adults at nutritional risk was 2.192-fold greater than in their well-nourished counterparts. Shawky et al. (63) found a higher frequency of MCI in those who were malnourished or at risk of malnutrition compared to those who were well-nourished, and the authors also found that a nutritional deficit and MCI were strongly associated after adjusting for potential confounders. Kishino et al. (64) also found that during a 2.5-year follow-up, poor nutritional status increased BPSD in those with MCI and early-stage AD. If MCI is detected early, it may be reversible, and healthcare providers should provide dietary health guidance to improve the nutritional status and ensure adequate nutrition intake in older adult individuals. The bidirectional relationship between cognitive impairment and malnutrition is intricate, suggesting that those with mental challenges are also more vulnerable to nutritional deficits (65, 66).

The most common strategy for preventing or treating malnutrition is using nutritional supplements to increase the individual’s oral intake (67). It is also essential to properly assess nutritional status and provide individualized and systematic or routine guidance to prevent malnutrition. In daily practice, assessments that may help avoid malnutrition can be based on regular weight control, daily energy intake monitoring, and the development of regular meal plans. This control appears crucial, especially in older adult individuals with cognitive impairment, as they may have difficulty expressing their wishes and needs regarding meals. To improve the health of older adults, older adults should pay attention to nutritional supplementation and lifestyle adjustment. Older adults need to consume sufficient protein and various micronutrients to maintain their normal physical and cognitive functions. At the same time, appropriate measures should be taken for the frail and cognitively impaired older adult, such as increasing nutritional intake, conducting rehabilitation training, and cognitive stimulation, to delay the progression of the disease. Lastly, health education and awareness efforts targeting older adults should intensify to bolster understanding of nutrition’s role and promote adopting a healthful lifestyle.

### 4.1 Strengths and limitations

This study’s findings offer valuable insights for community nurses aiming to mitigate the risk of MCI among older adults. A significant strength of this research is its large sample size, encompassing 706 older individuals from Southwest China, which enhances the generalizability of the results within this region. Nonetheless, the study has limitations. The cross-sectional design impedes the ability to infer causality. Additionally, as the research was confined to a single city, the findings may be applicable to other regions in China. Given these constraints, further research is imperative to corroborate these results and extend their applicability.

## 5 Conclusion

The study revealed a notable incidence of MCI among the senior population within the surveyed communities, particularly among those suffering from malnutrition. A significant link was established between malnutrition, as assessed by the Mini Nutritional Assessment-Short Form (MNA-SF), and cognitive decline. Specifically, malnutrition risk, advanced age, rural residency, male gender, intermediate education levels, and dependence on Activities of Daily Living (ADL) were identified as associated factors of MCI. Proactive early screening and tailored nutritional interventions are recommended to arrest cognitive deterioration in these susceptible demographics. Longitudinal investigations are warranted to delineate the temporal dynamics between MCI, demographic variables, and the potential mitigating effects of enhanced ADL or nutritional status on cognitive health in the older adult cohort.

## Data Availability

The raw data supporting the conclusions of this article will be made available by the authors, without undue reservation.
